# GDF‐5 induces epidermal stem cell migration via RhoA‐MMP9 signalling

**DOI:** 10.1111/jcmm.15925

**Published:** 2020-12-27

**Authors:** Xue Li, Fan Wang, Yuanxin Lan, Ruyu Bian, Ying Wang, Xiaorong Zhang, Yicheng Guo, Ling Xiao, Wenqiang Ni, Xiaohong Zhao, Gaoxing Luo, Rixing Zhan

**Affiliations:** ^1^ Institute of Burn Research State Key Laboratory of Trauma, Burn and Combined Injury, Key Laboratory of Proteomics of Chongqing Southwest Hospital The Third Military Medical University (Army Medical University) Chongqing China; ^2^ Department of Plastic and Reconstructive Surgery Southwest Hospital The Third Military Medical University (Army Medical University) Chongqing China; ^3^ Department of Burn and Plastic Surgery Chenzhou First People's Hospital Affiliated to Nanhua University Chenzhou China

**Keywords:** cell migration, epidermal stem cells, growth/differentiation factor‐5, matrix metalloproteinases

## Abstract

The migration of epidermal stem cells (EpSCs) is critical for wound re‐epithelization and wound healing. Recently, growth/differentiation factor‐5 (GDF‐5) was discovered to have multiple biological effects on wound healing; however, its role in EpSCs remains unclear. In this work, recombinant mouse GDF‐5 (rmGDF‐5) was found via live imaging in vitro to facilitate the migration of mouse EpSCs in a wound‐scratch model. Western blot and real‐time PCR assays demonstrated that the expression levels of RhoA and matrix metalloproteinase‐9 (MMP9) were correlated with rmGDF‐5 concentration. Furthermore, we found that rmGDF‐5 stimulated mouse EpSC migration in vitro by regulating MMP9 expression at the mRNA and protein levels through the RhoA signalling pathway. Moreover, in a deep partial‐thickness scald mouse model in vivo, GDF‐5 was confirmed to promote EpSC migration and MMP9 expression via RhoA, as evidenced by the tracking of cells labelled with 5‐bromo‐2‐deoxyuridine (BrdU). The current study showed that rmGDF‐5 can promote mouse EpSC migration in vitro and in vivo and that GDF‐5 can trigger the migration of EpSCs via RhoA‐MMP9 signalling.

## 1 INTRODUCTION

Wound healing, as a basic physiological process, is crucial for the maintenance of skin integrity. Multiple factors mediate the regeneration and reorganization of skin, which are complex and dynamic processes.^1^ However, the mechanisms of skin wound healing have not been fully elucidated. A variety of cellular molecules and components contribute to skin homeostasis and regeneration. EpSCs (Epidermal stem cells), which reside in the stratum basal of the epidermis and in the hair follicle wall, have essential roles in maintaining skin homeostasis and skin rehabilitation.^2^ Both interfollicular epidermal cells and hair follicle stem cell masses can regenerate the whole structure of skin amid skin injury. EpSCs migrate to the wound and then proliferate and differentiate in course of re‐epithelialization and reorganization.^3^ However, the mechanisms underlying the stimulation of EpSC migration during wound healing are unclear.

Growth/differentiation factor‐5 (GDF‐5), also known as cartilage‐derived morphogenetic protein‐1, is a member of BMP (bone morphogenetic protein) superfamily. GDF‐5 is crucial for mesenchymal cell differentiation and the morphogenesis of tendon, skeletal and ligament tissues.^4^ GDF‐5 activates the proliferation of human periodontal ligament cells. Members of the BMP family of dimeric proteins, which include GDF‐5, participate in bone and cartilage formation. In addition, BMPs play regulatory roles in many different cell types, influencing cell proliferation, differentiation, migration and apoptosis.^5^, ^6^ BMPs are expressed in a variety of tissues, including skin, where re‐epithelialization and reorganization are important steps in wound healing.^7^ Furthermore, BMPs regulate many signalling pathways, such as the pathways involving small GTPases (RhoA and Cdc42), extracellular signal‐regulated kinase, p38 mitogen‐activated protein kinase, phosphoinositide 3‐kinase‐Akt and c‐Jun N‐terminal kinase.^8^ Matrix metalloproteinases (MMPs) take part in extracellular matrix (ECM) degradation as the principal secreted proteinases during cell migration and metastasis.^9^ MMP9 is considered to enhance cell migration and has attracted substantial research attention. In addition, the RhoA pathway was shown to mediate the MMP9‐independent invasive behaviour of a triple‐negative breast cancer cell line.^10^

In the present study, we investigated the effect of GDF‐5 on primary mouse EpSC migration in a monolayer cell scratch model via live imaging, and we explored the possible mechanism with real‐time PCR assays, pull‐down assays and Western blot analysis in vitro. The promotion of EpSC migration and MMP9 expression by GDF‐5 via RhoA was confirmed in vivo in a line of 5‐bromo‐2‐deoxyuridine (BrdU)‐labelled cells from a deep partial‐thickness burn mouse model. Our experiments showed that in vivo and vitro, GDF‐5 enhanced EpSC migration through the RhoA/MMP9 signalling pathway.

## 2 MATERIALS AND METHODS

### 2.1 Materials, specimens and study approval

C57BL/6 mice were obtained from the Experimental Animal Department of the Third Military Medical University, China. The mice were housed individually in plastic cages under standard conditions (circadian rhythm of 12 hours, relative humidity of 50% and temperature of 25°C). The mice were provided normal autoclaved rodent chow and water ad libitum and allowed to acclimate to the facility for one week before the experiment was initiated. Each surgery was conducted with the mouse under 0.10% pentobarbital sodium anaesthesia. All efforts were made to minimize animal pain and suffering.

### 2.2 Characterization and culture of mouse EpSCs

Cells were isolated from the dorsal and ventral neonatal mice skin as described in our previous studies.^11^, ^12^, ^13^ Briefly, mice skin tissues were incubated with 0.25% Dispase II (04942078001; Roche) at 4°C overnight. Then, 10‐minute dissociation of the separated epidermis into single cells was performed with 0.25% trypsin at 37°C. At a density of 10^5^ cells/cm^2^, cells were seeded into flasks or onto plates coated with 100 μg/mL type IV collagen (C5533; Sigma) for a 10‐minute adherence period at 37°C. Non‐adherent cells were discarded, and adherent cells were cultured at 37°C in a 5% CO_2_ atmosphere in complete medium containing K‐SFM (17005; Gibco), mouse recombinant epidermal growth factor (0.1‐0.2 ng/mL; 315‐09‐100; Peprotech), bovine pituitary extract (20‐30 mg/mL), mouse epidermal growth factor (10 ng/mL; 354 001; BD), cholera toxin (1 × 10^−10^ mol/L; C9903; Sigma), calcium chloride (0.05 mmol/L) and streptomycin/penicillin solution (100 IU/L; Gibco; 15140122). The medium was changed every two days. The cells at the second passage were used for identification as well as for other experiments.

Cultured cells were subjected to flow cytometry analysis. Dual staining was carried out with phycoerythrin‐conjugated α6 integrin (555736; BD) and FITC‐conjugated CD71 (561936; BD). Flow cytometry was conducted with an Attune Acoustic Focusing Cytometer (Applied Biosystems, Life Technologies), and the data were analysed using FlowJo software (Tree Star Corporation).

### 2.3 Transient transfection of cells

Vector encoding dominant‐positive RhoA^(+/+)^ (RhoA G14V: valine was used instead of the glycine at residue 14) or dominant‐negative RhoA^(−/−)^ (RhoA T19N: asparagine was used instead of the threonine at residue 19) was used. pGFP control plasmid was obtained from Fisher Scientific.^14^ A total of 1.5 μg/mL pGFP control plasmid, 1.5 μg/mL RhoA^(−/−)^ (T19N) or 1.5 μg/mL RhoA^(+/+)^ (G14V) was transfected into mouse EpSCs using Lipofectamine 2000 (Invitrogen) in accordance with the manufacturer's protocol. Experimentation was performed after the cells had stabilized in complete medium for 24 hours.

### 2.4 Wound‐scratch assay

The migration of mouse EpSCs was evaluated by using a scratch wound model in vitro as described previously,^15^ with minor alternations. Briefly, mouse EpSCs were cultured to near confluence in 6‐well cell culture plates pre‐coated with type IV collagen. Mitomycin C (Sigma) was added to a concentration of 4 µg/mL, followed by cell incubation for 2 hours to halt cell proliferation.^16^ Next, a cell‐free area was created in each well by scraping away the cell monolayer with a sterile 10‐µL plastic pipette tip. The non‐adherent cells and medium were aspirated, and the plates were rinsed with PBS. Then, recombinant mouse GDF‐5 (rmGDF‐5, 315‐09‐100, Peprotech) at one of several concentrations was added along with fresh culture medium to the wells, with 3 wells per concentration. The wound was regularly imaged by inverted phase microscope over a twenty‐four hours period. The quantification of residual area among the migrating EpSCs was achieved by ImageJ software (NIH). The fractional closure of the scrape wound was calculated by the equation: (original area − remaining area)/original area × 100%.

### 2.5 Cell motility assay

Each one‐cell movement assay was carried out in 24‐well plates (Corning Costar) as described previously.^17^ Briefly, mouse EpSCs were seeded at 2 × 10^4^ cells/mL/well into 24‐well plates pre‐coated with type IV collagen and cultured for 48 hours under humid conditions with 5% CO_2_ at 37°C. Via time‐lapse video, a 24‐hour record of cell motility was obtained by a Zeiss Axiovert 135T inverted microscope. Images collected at five‐minute intervals were obtained with AQM Advance 6 Kinetic Acquisition Manager (Medical Solutions, PLC) and analysed using Adobe ImageReady software. Cell speed was calculated from hourly images of a 24‐hour recording of the cell periphery using ImageJ tracking software (NIH).

### 2.6 Western blotting

Western blot assays were carried out as described above. In short, proteins from lysates of skin epidermal tissue and stimulated cells were transferred to nitrocellulose membranes following electrophoreses on 10% SDS‐PAGE gels. The membranes were then blocked at room temperature with Tris‐buffered saline (TBS) containing 3% bovine serum albumin (BSA) and then incubated with anti‐GAPDH antibody (SAB2100894, Sigma, 1:800) or anti‐MMP9 antibody (GTX100458, GeneTex, 1:600) overnight at 4°C to evaluate the expression of target proteins. (The anti‐MMP9 antibody reacts with a 78‐kD active form of MMP‐9.) The membranes were then washed thrice with TBS containing 0.1% Tween‐20. After a one‐hour incubation with horseradish peroxidase‐bond second‐class antibody at room temperature, the membranes were washed again, and the bands were visualized through enhanced chemiluminescence.

### 2.7 Pull‐down assay

RhoA activity was measured using pull‐down assay kits (Cat. BK034, Cytoskeleton) following the manufacturer's instructions. Rhotekin‐RBD beads were used to indicate RhoA activity. The beads were washed with lysis buffer, and bound GTP‐Rho was detected through immunoblot analysis with rabbit anti‐mouse monoclonal RhoA antibody (1:400) and goat anti‐rabbit HRP‐conjugated secondary antibody (1:1000). RhoA signal was detected based on enhanced chemiluminescence.

### 2.8 RNA isolation and real‐time PCR

Total RNA was extracted by TRIzol reagent (Invitrogen), and one microgram of total RNA was reverse transcribed. Quantitative real‐time PCR (Q‐RT‐PCR) was implemented on a 7500 Real‐Time PCR System (Applied Biosystems) with SYBR Green to determine the mRNA expression levels of the genes of interest. Expression levels were normalized to GAPDH RNA expression. The primer sequences were as follows: RhoA: forward, 5′‐AATGACGAGCACACGAGACGGGA‐3′ and reverse, 5′‐ATGTACCCAAAAGCGCCAATCCT‐3′; MMP9: forward, 5′‐ACGGACCCGAAGCGGACATT‐3′ and reverse, 5′‐TTGCCCAGCGACCACAACTC‐3′; GAPDH: forward, 5′‐CGTGCCGCCTGGAGAAAC‐3′ and reverse, 5′‐AGTGGGAGTTGCTGTTGAAGTC‐3′.

### 2.9 Immunocytochemistry

Mouse EpSCs were seeded onto coverslips pre‐coated with type IV collagen, fixed with 70% methanol in acetone and blocked for 1 hour with BSA 1% (Sigma) at ambient temperature. The cells were then incubated with rabbit anti‐mouse MMP9 (GTX100458, GeneTex, 1:400) at 4°C overnight, followed by a one‐hour incubation with phycoerythrin (PE)‐labelled donkey anti‐rabbit second‐class antibody (1:500; Invitrogen) under the same conditions and a five‐min incubation with DAPI (5 μg/mL, Sigma). The stained cells were examined under a laser scanning confocal fluorescence microscope (Leica).

### 2.10 MMP9 expression in a deep partial‐thickness burn model

The labelling of EpSCs in mouse skin with 5‐bromo‐2′‐deoxyuridine (BrdU) and the establishment of a deep partial‐thickness burn model were performed as described previously.^12^, ^13^ In short, neonatal C57BL/6 mice were intraperitoneally injected with BrdU (50 mg/kg body weight, B5002, Sigma) twice a day for three days at three days of age. The mice were then maintained for 7 weeks to allow sufficient time for skin cells to be identified as EpSCs. The mice were then anaesthetized with an i.p. injection of 0.1% sodium pentobarbital at 10 µL/g of body weight, and interscapular hair removal was performed. After that, a metal plate (Shandong Academy of Medical Science, Jinan) with a 1.5‐cm diameter and a 0.5‐kg weight was used to induce deep local‐thickness burns. The metal plate was heated to 70°C and then placed for 3 seconds on the shaved mouse dorsum. Each mouse was housed individually in a plastic cage under standard conditions.

The injured mice were randomly divided into three groups: normal saline (NS, 0.9% sodium chloride as a control), rmGDF‐5 and rmGDF‐5 + C3 transferase (RhoA antagonist). Immediately after injury, the mouse was injected (0.05 mL/g body weight, i.p.) with rmGDF‐5 (10 µg/mL in NS), C3 transferase (60 µg/L, Sigma), or NS. Every group comprised 6 mice. After 48 hours, each mouse was anaesthetized with an i.p. injection of 0.1% sodium pentobarbital in a volume of 10 µL/g of body weight and then euthanized by cervical dislocation. The wounds were biopsied, and the samples were fixed with paraformaldehyde and then sectioned. Immunofluorescence staining was performed to detect BrdU (B8434, Sigma) and MMP9 (GTX100458, GeneTex). Under a microscope at 400× magnification, 6 fields per section were randomly imaged. The number of BrdU^+^MMP9^+^ cells in the re‐epithelialization area of every field was counted using Image‐Pro Plus software (Media Cybernetics).

### 2.11 Statistical analysis

The data are presented as the means ± SD. Comparisons between groups were performed via Student's *t* test. One‐way analysis of variance (ANOVA) was used to test for differences among groups. A *P* value < .05 was considered to indicate statistical significance.

## 3 RESULTS

### 3.1 Effect of GDF‐5 on the migration of isolated mouse EpSCs in vitro

**Figure 1:**
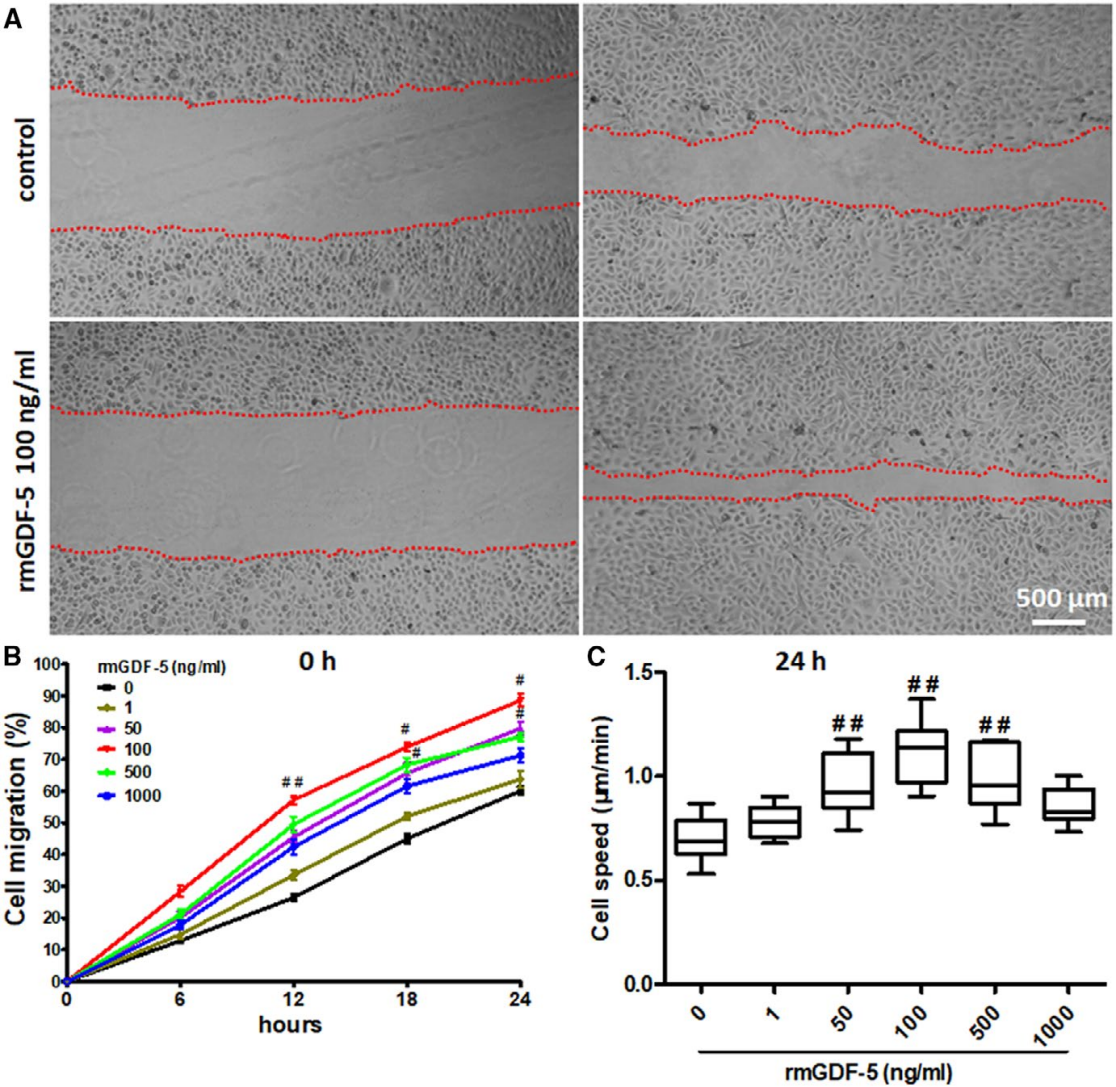
GDF‐5 promotes mouse EpSC migration in vitro. rmGDF‐5 stimulated artificial wound closure. Wounds were established in merging monolayers of mouse EpSCs as described in the materials and methods section. A, The wound edges were time‐lapsed imaged under an inverted phase‐contrast microscope. The residual area was quantified using ImageJ software. Scale bar, 500 µm. B, Data from the images shown in (A). Migration rate was calculated using the following equation: (original area − remnant area)/original area × 100%. C, Average cell speed was determined from hourly images tracking the cell periphery. We used ImageJ tracking software (NIH) to measure cell speed. The results are presented in a box plot; the line indicates the median speed, and the minimum and maximum values are marked by whiskers. Data represent the values from 3 independent experiments (one‐way ANOVA); ^##^ *P* < .01 and ^#^ *P* < .05 compared with the control (0 ng/mL rmGDF‐5 as control)

EpSCs were isolated from neonatal mice as described in previous studies.^11^, ^12^, ^13^, ^18^ The EpSC‐specific markers α6 integrin and CD71 were detected by flow cytometry. Flow cytometry indicated rich α6 integrin expression and minor CD71 expression of the cultured cells (Figure S1). Migration of the isolated mouse EpSCs was detected by wound‐scratch model and time‐lapse cell motility assays. In the scratch‐wound model, a dose‐dependent increase in cell migration with rmGDF‐5 was observed over a concentration range of 1‐100 ng/mL. The concentration of 100 ng/mL can be considered the optimum rmGDF‐5 concentration for inducing cell migration (Figure 1B). As shown in Figure 1C, a time‐lapse cell motility assay was carried out with single cells. Cell motility changed with rmGDF‐5 exposure in a dose‐dependent manner. Twenty‐four‐hour exposure of EpSCs to rmGDF‐5 (1‐100 ng/mL) significantly enhanced EpSC motility, and the optimal rmGDF‐5 concentration for inducing cell migration was 100 ng/mL. These findings indicated that 100 ng/mL rmGDF‐5 was the optimal concentration for inducing mouse EpSC migration.

### 3.2 GDF‐5 regulates the mRNA and protein levels of RhoA and MMP9

**Figure 2:**
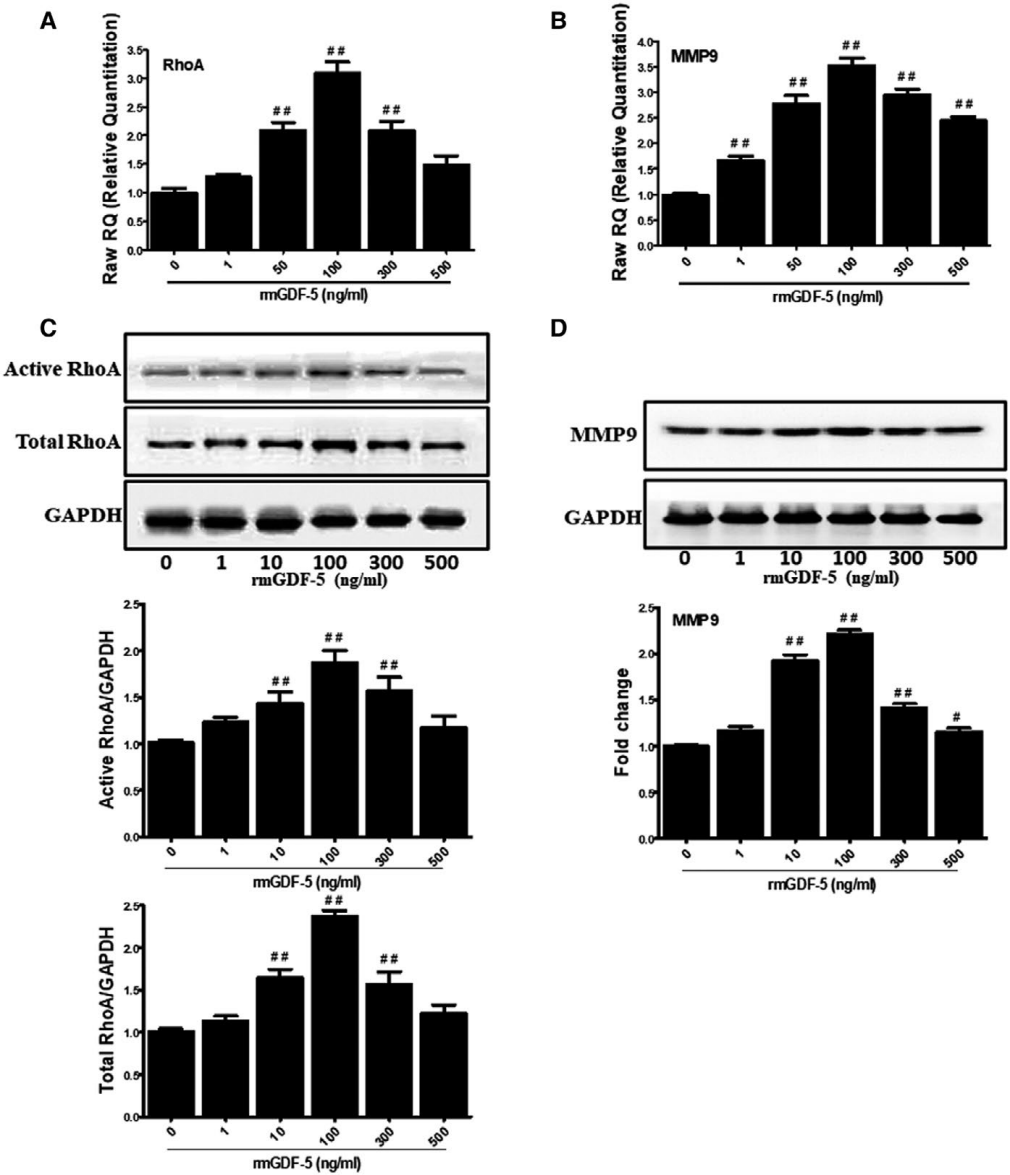
GDF‐5 induces the expression of RhoA and MMP9 in a dose‐dependent manner. EpSCs of mice lacking growth factor were treated with rmGDF‐5 at concentrations ranging from 0 to 500 ng/mL for 24 h. A and B, The mRNA expression of RhoA and MMP9 was determined using real‐time PCR. C, Active RhoA was detected with Rhotekin‐RBD beads as described in the section Materials and methods. RhoA loaded with GTP and total RhoA were detected using Western blotting, and the band intensities of the indicated proteins were detected through densitometry. D, MMP9 protein levels were evaluated using Western blotting, and the band intensities of the indicated proteins were quantified through densitometry. The data represent the mean ± SD of 3 independent experiments. The significance of differences was evaluated via unpaired Student's *t* test (two tailed): ^##^ *P* < .01, ^#^ *P* < 0.05 compared with the control (0 ng/mL rmGDF‐5 as the control)

We evaluated the protein and mRNA levels of RhoA and MMP9 to investigate whether the rmGDF‐5‐induced migration of EpSCs is mediated by RhoA and MMP9. As shown in Figure 2A,B, mRNA expression of RhoA and MMP9 was induced by rmGDF‐5 in a concentration‐dependent manner, and 100 ng/mL rmGDF‐5 was identified as the optimal concentration for inducing RhoA and MMP9 mRNA expression. In Western blot and pull‐down assays, the levels of active and total RhoA protein and the levels of pro and active isoforms of MMP9 were increased by rmGDF‐5 in a concentration‐dependent manner (Figure 2C,D). In addition, 100 ng/mL rmGDF‐5 was the optimal concentration for inducing RhoA and MMP9 protein expression. Thus, the expression levels of RhoA and MMP9 protein and mRNA were correlated with rmGDF‐5 concentration, and the optimal concentration for inducing expression was 100 ng/mL. These results show that the effects of GDF‐5 on RhoA and MMP9 were achieved through transcription‐level regulation.

### 3.3 GDF‐5 stimulates mouse EpSC migration via RhoA‐MMP9‐mediated signal transduction

The transfection efficiencies of the RhoA^(−/−)^ (T19N), RhoA^(+/+)^ (G14V) and pGFP control plasmids varied from 90% to 98% over the 24‐hour transfection period, as determined by GFP fluorescence (Figure S2A), revealing the successful transfection of each plasmid into mouse EpSCs. Additionally, the changes in RhoA activity caused by constitutively active (G14V) RhoA mutants were confirmed by a RhoA pull‐down assay (Figure S2B). The RhoA activity induced by G14V RhoA mutants was 2.08‐fold above that of the control.

**Figure 3:**
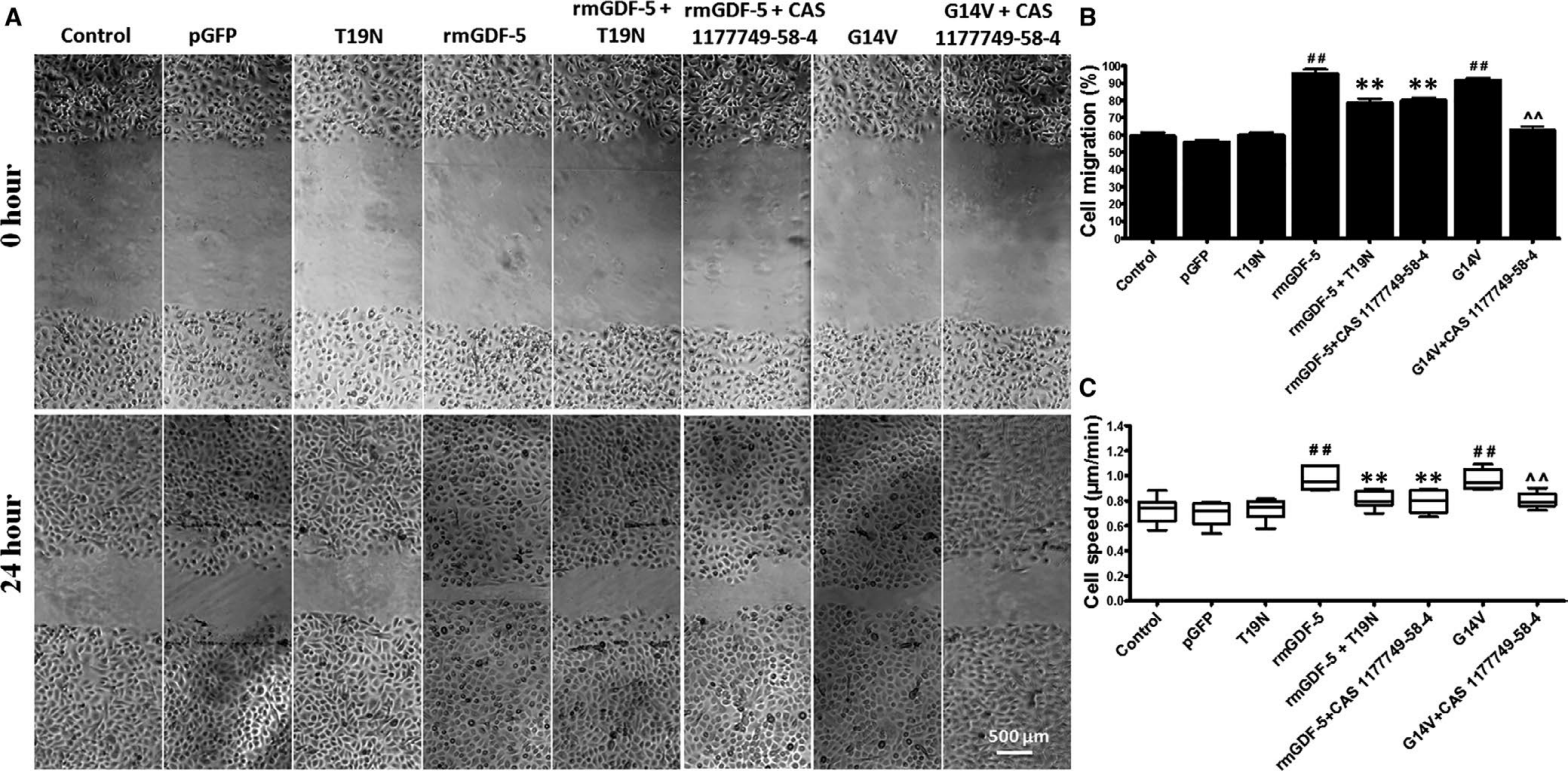
GDF‐5 regulates mouse EpSC migration in vitro via RhoA‐MMP9 signalling. Using Lipofectamine transfection reagent, mouse EpSCs were transfected with pGFP control plasmid, RhoA^(+/+)^ (G14V) or RhoA^(−/−)^ (T19N) for 48 h. Additionally, cells transfected with T19N plasmid were treated with 100 ng/mL rmGDF‐5 or 5 nmol/L CAS 1177749‐58‐4 (a MMP9‐specific inhibitor), and cells transfected with G14V plasmid were treated with or without 5 nmol/L CAS 1177749‐58‐4. A, Wounds were established in merging monolayers of mouse EpSCs, and the wounds were monitored for 24 h following treatment. Images of the wound edges were recorded at regular intervals under an inverted stage microscope. The data represent the results of 3 independent experiments. Scale bar, 500 µm. B, Data from of the images shown in (A). Cell migration was quantified and displayed as a function of delayed time vs percent of wound gap. C, Cell motility assay was carried out as described in the section Materials and methods. The values are the means ± SD of 3 independent experiments; the significance of differences was assessed using unpaired Student's *t* tests (two tailed); ^##^ *P* < .01, ^#^ *P* < .05 compared with the control; *P < .05, **P < .01 compared with the rmGDF‐5 group; and ^^^ *P* < .05, ^^^^ *P* < .01 compared with the G14V group

After transfection, mouse EpSCs were treated with different reagents and subjected to scratch assays (Figure 3A) and single‐cell motility assays (Figure 3C) to determine whether cell migration was facilitated by rmGDF‐5 through a potential target molecule downstream. The 100 ng/mL rmGDF‐5 treatment significantly increased EpSC migration and motility (*P* < .01), and these effects were markedly reduced by the T19N plasmid or the MMP9 inhibitor CAS 1177749‐58‐4. Furthermore, the group expressing the RhoA^(+/+)^ plasmid G14V exhibited significantly higher EpSC migration rates than the control group, and this influence of G14V was inhibited by the MMP9 inhibitor CAS 1177749‐58‐4. These results indicate that rmGDF‐5 stimulates mouse EpSC migration via the RhoA‐MMP9 signalling pathway.

### 3.4 GDF‐5 up‐regulates MMP9 expression via RhoA in vitro

**Figure 4:**
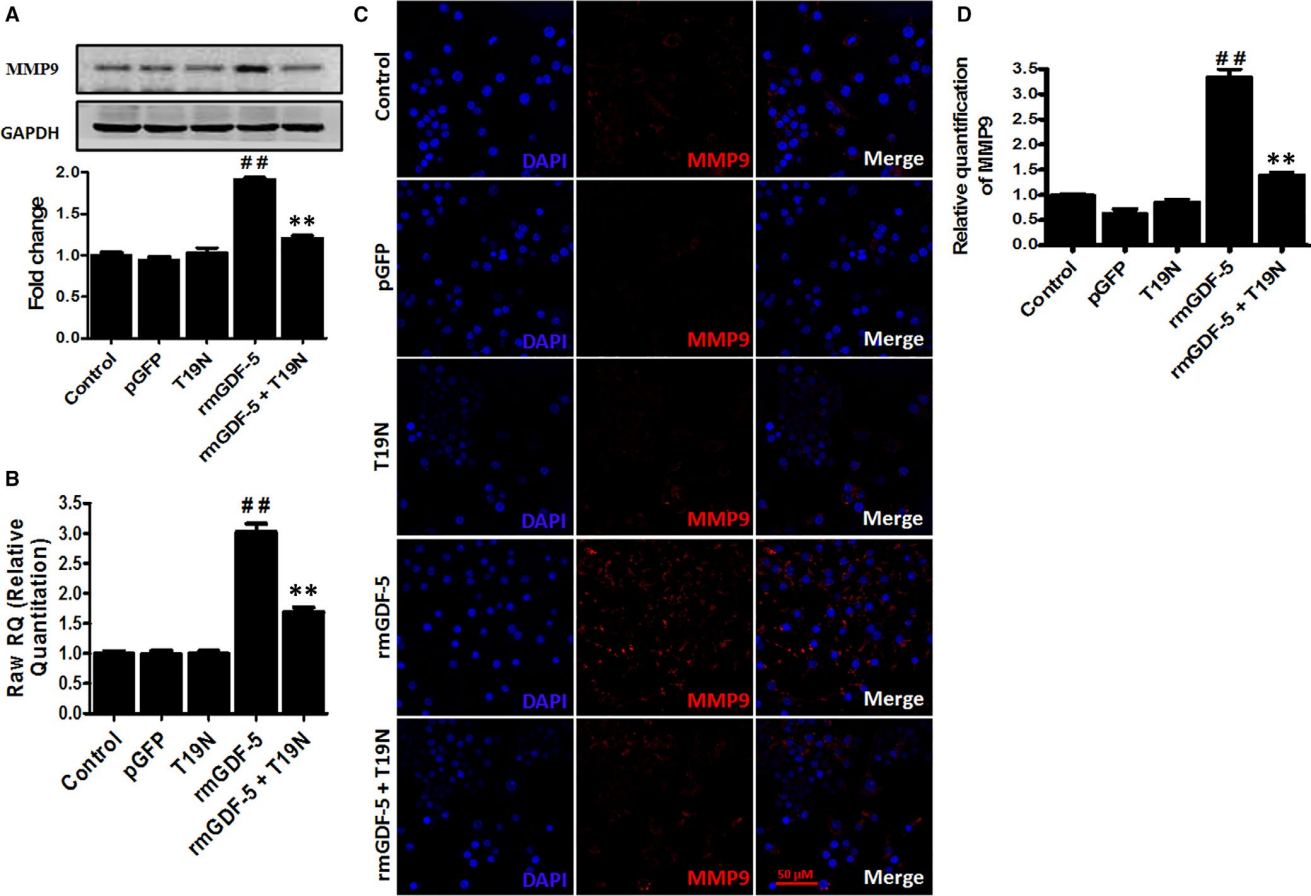
GDF‐5 regulates the expression of MMP9 through RhoA signalling in vitro. Mouse EpSCs lacking growth factor were stimulated with 100 ng/mL rmGDF‐5 following transfection with RhoA^(−/−)^ T19N or control plasmid. A, The active and pro isoforms of MMP‐9 were detected using Western blotting, and band intensity was quantified using densitometry. B, MMP9 mRNA expression was measured using real‐time PCR, as described in the section Materials and methods. C, For immunohistochemical analysis, cells were stained with MMP9 antibody (red), and nuclei were stained by DAPI (blue). Scale bar, 50 μm. D, Image‐Pro Plus software was used for data analysis. The corresponding IOD for MMP9 in EpSCs is shown. Data are the means ± SD of 3 independent experiments; the significance of differences was assessed using unpaired Student's *t* tests (two tailed): ^##^ *P* < .01 compared with the control group; *P < .05, **P < .01 compared with the rmGDF‐5 group

The mRNA and protein levels of MMP9 were determined by Western blotting, cellular immunofluorescence and real‐time PCR to evaluate the relationships among GDF‐5, RhoA and MMP9. Higher levels of the pro and active isoforms of MMP9 were detected in the rmGDF‐5 group than in the control group (*P* < .01). Furthermore, the expression‐inducing effect of rmGDF‐5 was inhibited by the RhoA^(‐/‐)^ T19N plasmid (Figure 4A). Real‐time PCR assays revealed significantly up‐regulated expression of MMP9 mRNA in the rmGDF‐5 group compared with the control group (*P* < .01); this up‐regulation was blocked by the RhoA^(−/−)^ T19N plasmid (Figure 4B). In the cellular immunofluorescence assay, higher fluorescence intensity of the MMP9 protein was observed in rmGDF‐5 group than the control group; this enhancement was inhibited by RhoA^(−/−)^ T19N plasmid (Figure 4C,D). These results indicate that GDF‐5 up‐regulates MMP9 expression via RhoA, potentially at the transcriptional level.

### 3.5 GDF‐5 promotes EpSC migration and MMP9 expression via RhoA in vivo

The mouse deep partial‐thickness scald model was constructed with BrdU‐labelled EpSCs in vivo as described in previous studies^13^, ^19^ to assess the effects of rmGDF‐5 on MMP9 expression and EpSC migration in vivo. For the in vivo labelling of mouse EpSCs, which presented largely as label‐retaining cells (LRCs) in skin, new‐born mice were subcutaneously injected with BrdU. After a 6‐8 week period, the BrdU‐LRCs had developed into EpSCs (Figure S3). The findings agree well with previous studies using BrdU and ^3^H‐thymidine (^3^H‐TdR), indicating that the skin cells labelled with BrdU were predominantly bulge EpSCs.^20^, ^21^ After the BrdU‐LRCs had developed for 7 weeks, deep partial‐thickness burns were established, and after 48 hours, the migration of EpSCs was analysed by determining the number of BrdU‐LRCs in the regenerated cutex. In this model, EpSC migration is complex process accompanied by both proliferation and differentiation.

**Figure 5:**
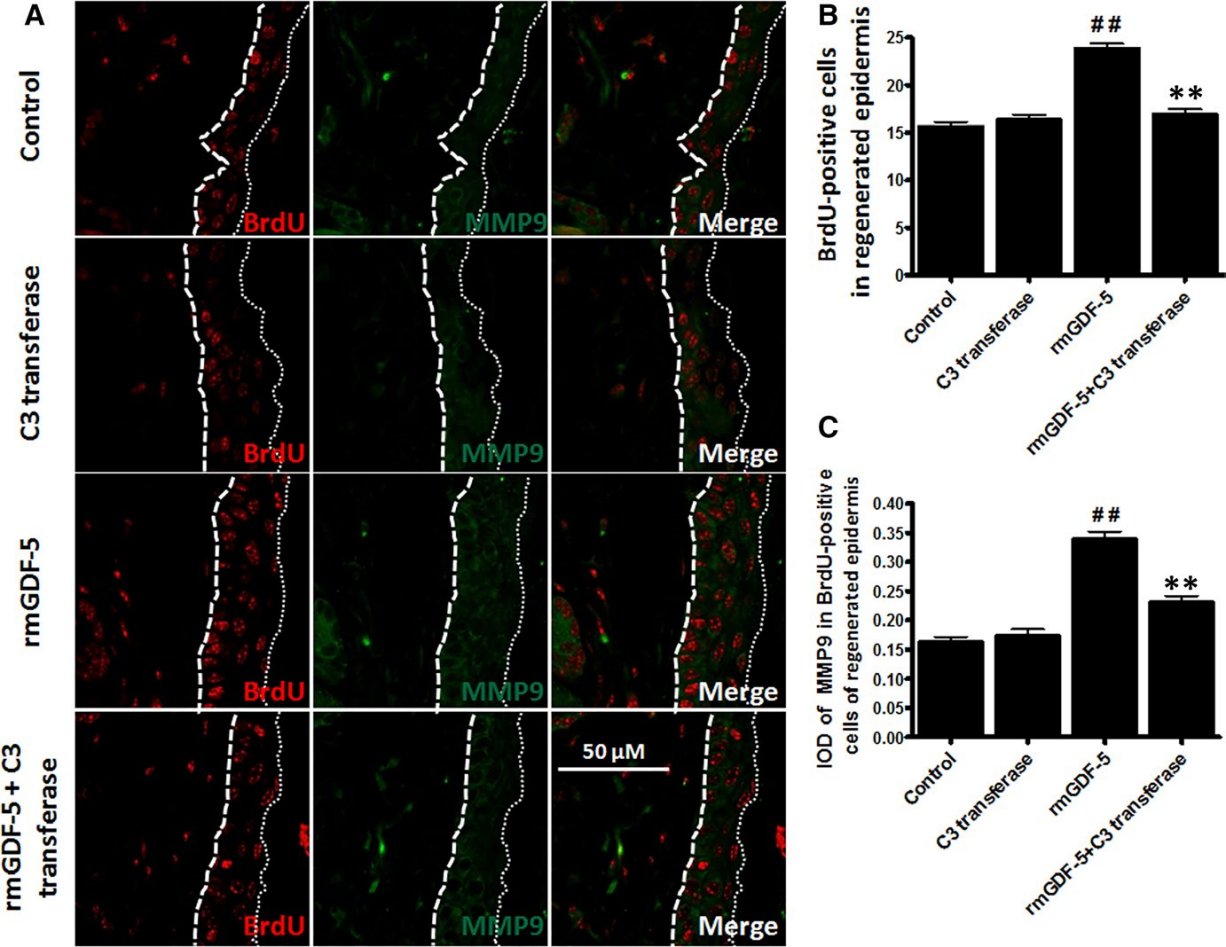
Immunohistochemical staining for MMP9 in cells labelled with BrdU. A, Immunohistochemical staining of BrdU in cells of mice treated with NS (normal saline as a control), C3 transferase (RhoA antagonist), rmGDF‐5 or rmGDF‐5 + C3 transferase. Images of BrdU (red) staining were captured at 400× magnification. Same‐magnification representative images show cells of complete formation. The two dotted lines represent the regenerated cutex. B and C, Data were analysed using Image‐Pro Plus software. B, Columns indicate the average number of cells positive for BrdU in the regenerated cutex. C, The integrated optical density (IOD) of MMP9 in BrdU‐positive cells in the regenerative cutex is shown. The data are the means ± SD of 3 independent experiments (unpaired, two‐tailed Student's *t* tests); ^##^ *P* < .05 compared with the control group, **P < .01 compared with the rmGDF‐5 group

As shown in Figure 5, there was a marked difference in the number of cells positive for BrdU in the regenerated cutex between the rmGDF‐5 group and the control group. An increase in the number of cells positive for BrdU occurred with rmGDF‐5 treatment, and this increase was prevented by the RhoA inhibitor C3 transferase (*P* < .01, Figure 5A,B). Additionally, a higher integrated optical density (IOD) of MMP9 in BrdU‐positive cells in the regenerated cutex (between the two dotted lines) was observed in the GDF‐5 group than in the control group. Additionally, the IOD of MMP9 in BrdU‐positive cells in the GDF‐5 group much higher than that in the control group (*P* < .01). In addition, the increase in IOD was suppressed by RhoA inhibitor C3 transferase (*P* < .01, Figure 5A,C). These findings indicate that GDF‐5 promotes EpSC migration through RhoA‐MMP9 signalling in vivo.

## 4 DISCUSSION

Although several studies of the effects of GDF‐5 on wound healing have been performed,^22^, ^23^, ^24^ the mechanisms remain unclear. The migration of EpSCs is the most critical process in wound re‐epithelization and wound healing. In this study, we found that rmGDF‐5 could promote mouse EpSC migration in vivo and in vitro and that GDF‐5 accelerated EpSC migration via RhoA‐MMP9 signalling.

The BMP family of dimeric proteins, including GDF‐5, participates in bone and cartilage formation. In addition, the migration, proliferation, differentiation and apoptosis of various types of cells are regulated by BMPs. BMPs are expressed in different tissues, including skin,^25^ and EpSC migration and mobility are important steps in skin wound healing. Researchers have reported that GDF‐5 accelerates wound closure during the repair of full‐thickness skin defects.^24^ GDF‐5 represents a new therapeutic agent for periodontal wound healing/regeneration,^26^ and GDF‐5 promotes fibroblast migration.^27^, ^28^ We found that exposure of EpSCs to rmGDF‐5, which is involved in wound healing, significantly increased EpSC motility and migration in the present study, and 100 ng/mL rmGDF‐5 was the optimal concentration for inducing cell migration (Figure 1). GDF‐5 is a growth factor, and ‘GDF‐5 should be considered when developing composite biomaterials for wound healing’ as indicated.^24^

The signalling pathways by which GDF‐5 regulates cell migration have not been elucidated previously. We found that RhoA and MMP9 expression correlated with rmGDF‐5 concentration and that 100 ng/mL rmGDF‐5 was the optimal concentration for inducing RhoA and MMP9 expression (Figure 2). We also found that the effects of GDF‐5 on RhoA and MMP9 were achieved through transcriptional‐level regulation. BMPs/GDFs have been reported to regulate several pathways, including the FAK, paxillin, RhoA, MAPK, NF‐κB and MMP pathways.^29^, ^30^, ^31^ The small GTPase RhoA is activated by guanine nucleotide exchange factors.^32^ Several biochemical, cellular and physiological studies have revealed that RhoA tightly regulates the formation of actin structures^33^ and cell migration.^34^, ^35^ In addition, several studies have described the biological roles of MMPs in a wide range of cellular processes, including metastasis, angiogenesis, host defence, migration, cancer invasion and proliferation.^36^, ^37^ Among the MMPs considered to increase cell migration, MMP9 was the focus of our present study. In addition, in a triple‐negative breast cancer cell line,^10^ type VIII collagen signalling via beta1 integrin, MMP expression and smooth muscle cell migration were found to be mediated by the RhoA pathway. Furthermore, the RhoA pathway has been reported to mediate MMP9‐independent invasive behaviour.^38^ By using RhoA^(−/−)^ (T19N), RhoA^(+/+)^ (G14V) and pGFP control plasmid transfection, a wound‐scratch model (Figure 3A) and single‐cell motility assays (Figure 3C), we found that rmGDF‐5 stimulates mouse EpSC migration via the RhoA/MMP9 signalling pathway in vitro. Additionally, we found that GDF‐5 up‐regulates MMP9 expression via RhoA, potentially at the transcriptional level (Figure 4). Moreover, using BrdU label‐retaining and deep‐partial thickness scald assays, we found that rmGDF‐5 promoted EpSC migration and regulated MMP9 expression via RhoA signalling in vivo (Figure 5).

In conclusion, we report that rmGDF‐5 can promote EpSC migration in vitro and that 100 ng/mL rmGDF‐5 is the optimal concentration for achieving this effect. EpSC migration plays in important role in wound healing. Additionally, rmGDF‐5 promotes EpSC migration in vivo, as evidenced from the EpSC migration model and the tracking of BrdU‐marked LRCs in deep local‐thickness burns. Furthermore, the mechanism underlying rmGDF‐5‐induced EpSC migration involves RhoA‐MMP9 signalling.

## CONFLICT OF INTERESTS

The authors have no competing interests to declare.

## AUTHOR CONTRIBUTIONS

**Xue Li:** Methodology, Writing‐original draft. **Fan Wang:** Methodology, Investigation; Visualization, Writing‐review & editing. **Yuanxin Lan:** Methodology, Writing‐original draft. **Ruyu Bian,** and **Ying Wang:** Investigation, Visualization. **Xiaorong Zhang,** **Yicheng Guo,** and **Ling Xiao:** Investigation, Formal analysis. **Wenqiang Ni,** **Xiaohong Zhao:** Investigation, Resources. **Rixing Zhan,** **Gaoxing Luo:** Conceptualization, Project administration, Writing‐review & editing.

## ETHICAL APPROVAL AND CONSENT TO PARTICIPATE

All animal experimental procedures were approved by the Committee on the Ethics of Animal Experiments of the Third Military Medical University. This work was performed in strict accordance with the recommendations in the Guide for the Care and Use of Laboratory Animals of the National Institutes of Health (NIH).

## CONSENT FOR PUBLICATION

All authors provided consent for publication.

## Supporting information

Supplementary Material

## Data Availability

All data generated and/or analysed during this study are included in this published article.
