# Supplementary material for: GDF‐5 induces epidermal stem cell migration via RhoA‐MMP9 signalling
Source: J Cell Mol Med. 2020 Dec 27;25(4):1939–48. doi: 10.1111/jcmm.15925 (PMC7882973; doi:10.1111/jcmm.15925)
Supplement: Supplementary file 1 — Supplementary Material [file JCMM-25-1939-s001.docx]

**Supplementary materials:**


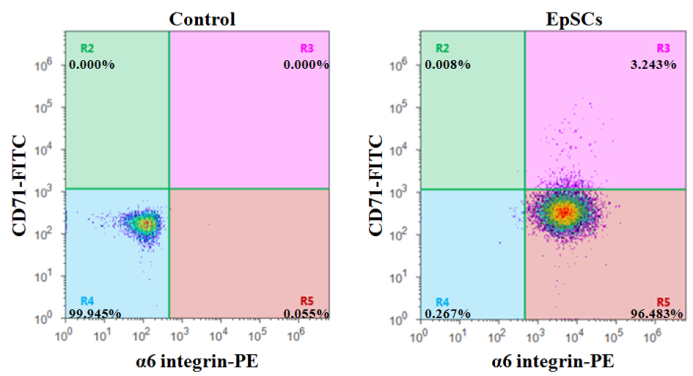


**Supplementary Figure 1.** The EpSC-specific marker α6 integrin was positively expressed, and the EpSC-negative marker CD71 was detected by flow cytometry.


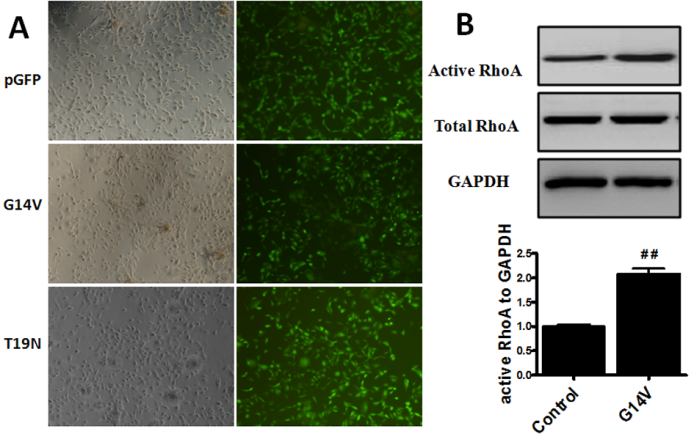


**Supplementary Figure 2. Plasmid transfection and detection of the RhoA^(+/+)^(G14V) plasmid.** RhoA^(+/+)^ (G14V), RhoA^(-/-)^ (T19N) or pGFP control plasmid (1.5 μg/ml of each plasmid) was transfected into mouse EpSCs by Lipofectamine transfection reagent. (A) Phase-contrast and pGFP fluorescent micro-graphs are shown (40× magnification). (B) Cytolysate was collected, and the amounts of active and total RhoA were analysed by pull-down assay. Protein quantification of blots was performed via densitometry. The data are plotted as the ratio of active RhoA to GAPDH; control: mice without EpSC transfection. **P<0.01 vs. the control group. Data are the mean±SD of replicate samples from 3 donors (unpaired two-tailed Student's t-test).


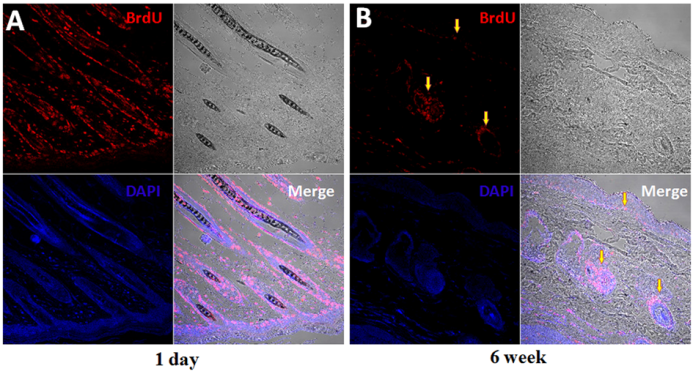


**Supplementary Figure 3. Immunofluorescence histochemistry of cells labelled with BrdU.** (A) One paraffin-cut section of skin from a mouse at three days of age that had undergone subcutaneous injection with BrdU twice a day for 3 days. The whole hair follicle and each epidermal cell of the stratum basale are labelled. (B) A paraffin section of the labelled mouse skin described in (A) tracked for six weeks. BrdU was selectively retained through a few cells of the cutex and the bulge cells. The arrow indicates positive staining.
